# Valedictory Address at Commencement Atlanta Medical College

**Published:** 1875-04

**Authors:** J. H. Logan

**Affiliations:** Professor of Chemistry in the College


ATLANTA

### Medical and Surgical Journal.

Vol. XIII.] APRIL—1875. [No. 1.

### Original Communications.

### VALEDICTORY ADDRESS.

*Delivered at the Annual Commencement of the Atlanta Medical College, 4th March, 1875. By J. H. Logan, M.D., Professor of Chemistry in the College.*

Gentlemen: It is with mingled feelings of pléasure and pain that I undertake to-night, by the appointment of my colleagues, to discharge the duties of valedictory speaker. It is always sad to say farewell to those with whom we have long held pleasurable social intercourse; and yet more sad when that intercourse has been consecrated by mutual sympathies and devotion in the cause of knowledge and truth. But, not unlike Uncle Toby, of Tristram Shandy, whose garrulous old age was cheered by living over again with his young companions the battles of his youth, I assure you it exhilarates me to stand with you on this occasion, so profoundly interesting to you as the threshold of your active life, that I may bid you a hearty God speed, and live with you again, in this hour, the bright dreams and fond anticipations of my own commencement day.

And ere we part, young gentlemen, I would seize the opportunity, so auspicious to us all, not for quaffing with you the rollicking poet's double health of wine, but to impress upon you a few well-tried principles of wisdom, that may serve you as friendly beacons amid the shadows and perplexities of your future career.

First of all, I would have you to realize, in all their fulness and meaning, the responsibilities you are about to assume as regularly constituted members of the medical profession. To be thus endowed, and full-furnished with the knowledge and talents equal to its demands, constitutes one of the rarest of those physical and mental combinations which mankind have ever honored with the name and attributes of greatness. The highest type of human benevolence is the consecration of one's powers to the good of his fellow-men. It is this, and not paltry wealth, nor family influence, nor even genius alone, in whose memory the carved marble and imperishable bronze speak in every civilized land, pointing, meanwhile, to the sublimer monuments of love and veneration enshrined in the grateful hearts of posterity.

Of all the secular professional activities of men there is none more useful or more exalted than that of the healing art. It subsidizes alike all the energies of the intellect and well nigh all the resources of nature. To be a great physician is to be one of the gods. The superstition even of an age of civilized paganism dedicated splendid temples to his worship, and the great Master, the Saviour of mankind, while laying deep the foundations of Christianity on earth, consecrated for all times and all civilizations, in his own divine person as the great Physician, the true dignity of Medicine. Indeed, so closely allied are the two great professions of medicine and the gospel ministry, that we are almost inclined to demand for the first the divine call, so essential to the last. At all events, whether they always get them or not, they both demand the highest style of man.

The very fact, gentlemen, that you have made choice of this profession for your life's work is strong *prima facie* evidence of the consciousness in yourselves of both intelligence and moral worth; for no man, having any proper notions of its responsibilities and arduous duties, could, for one moment, seek to assume them unless largely conscious of that peculiar fitness. A base, sordid man, devoting himself to the charities, amenities and self-sacrificing life of the faithful physician, would be an act as incongruous as the gathering of grapes from thorns, or of figs from thistles.

Now it is one of the first laws of moral philosophy that enlarged responsibilities imply a corresponding increase of intelligence and wisdom. To assume them without this concomitant is an exhibition of that fool-hardy rashness which ventures to intrude where angels fear to tread. Hence it is that the measure of a physician's duties becomes the measure of his fitness and his influence. The first is an inherent element of his calling; the second is, in most part, supplied by his own industry and energy. Dispel, therefore, at once and forever, from your minds, the bootless notion that the completion of your college curriculum, and the attainment of your diplomas, are the goal of your professional studies. You have but laid—roughly, it may be, but surely—the foundation walls of your medical education, which may be justly compared to a temple, whose beautiful superstructure with classic pediment and entablature, enriched and perfected in all its parts, is yet an embryo thought, nestling deep down in the ideal possibilities of each one of you.

The largest and best work of the practice of medicine is purely intellectual, and no intellectual work is of any worth that does not come of earnest study. Laborious, unremitted studiousness has been characteristic of the great men of all professions in all ages. Mr. Calhoun was accustomed to say that if he differed intellectually from other men, it was because he surpassed them in the ability to study; and to the last active day of their lives neither he nor Mr. Webster would ever venture to speak on any important subject without previous careful preparation. If these great men found it necessary, even late in life, to study in order to discharge their duties as statesmen, what must be our obligations of the same kind, that we may conscientiously and successfully combat the thousand mental and physical ills to which flesh is heir.

But besides this higher moral incentive is the scarcely less valuable incentive of knowledge itself. Knowledge, like money, begets a thirst for more knowledge, and to multitudes its pursuit has been a lifelong delight. It is related of the famous Dr. Philip Syng Physick, of Philadelphia, that on all occasions when his favorite branch was not interested, he appeared dull and listless, but the moment surgery became the topic, or he had work to perform, his eye would kindle with its wonted fire, and quickly he was all aglow with enthusiasm. This love of knowledge for its own sake is especially fascinating when it allures to new or inexhausted fields of research. Medicine still abounds in these, and she beckons you to her help in their exploration. In your preliminary studies you have been gathering general principles, and learning the names of a few of the instruments with which you are to work, and in whose use you are, by and by, to become skilled; but there are other principles, and facts innumerable, besides those, which are yet to be discovered, defined and classified, from every department of the broad field of your profession. It will be your privilege to take an active part in this work of discovery and classification. You owe it to your country, to your fellow-men, and to yourselves as well, to do all in your power for the advancement of the profession of your choice. Nothing purely selfish or meanly illiberal should ever mingle with the spirit with which you assume the duties of the noble calling to which you have consecrated your lives.

The ages of superstition, with their effete despotisms, have long ago passed away, when men, regarded and moved only in mass as so much machinery, were wholly ignored in their individual powers and capabilities. Now the mass is nothing unless it is the aggregate force of trained individuality. Every man, however unpretending he is, may be a power in his sphere, and is expected to accomplish something in the grand work of renovating the world. True greatness does not consist in the dignity and magnitude of the work which may be assigned a man, but essentially in the motives and energy with which it may perform it. Were two angels, it has been beautifully said, sent from heaven to earth, the one to rule an empire and the other to sweep the streets of a filthy city, the latter would labor at his task as cheerfully as the other, and be no less great in his own eyes and those of all created intelligences. *Duty*, therefore, and not *human applause*, should be the motto of your professional life.

Your noble calling, embracing, as it does, so wide a field of learning and research, so much that is minute and delicate and mysterious and even heroic in the contingencies of human life, exacts of you correspondingly high and peculiar qualifications of heart and mind and person as well; it subsidizes not only all the attributes of the philosopher, but equally those of tho Christian and the gentleman. But we must be more explicit. There is one power which you should cultivate with assiduous care. I shall venture to call it here the *medical sense*, for it is in great part peculiar to the physician, embracing all and much more of that intense consciousness of the relations of cause and effect in the phenomena of nature and society, which rendered Newton and Gallileo the greatest of natural philosophers, and Shakspeare the prince of dramatists. It is something beyond the reach of mere philosophy, for those great thinkers would doubtless have made a poor figure as medical practitioners, lacking, with all their genius, a certain practical ability, which, more than all others, has characterized, as a class, English and American physicians. Science gives brilliancy, the medical sense adds practical success to brilliancy. In its last analysis it is the sagacity of common sense, brought to bear upon the diagnosis and treatment of disease. This is indeed a familiar phrase, but the faculty it represents is, perhaps, in its highest manifestation, as rare as the philosophic genius. No culture of the schools can impart it, and no privation of culture can wholly suppress it. Endowed with this sense, and with intellect trained to its utmost capacity, man goes forth, at once, a giant. Such characters were Cullen, and Mead, and Sydenham, and John Hunter. But if education cannot create this power, it can, nevertheless, greatly strengthen and increase it, when it is feebly present. It is to this that I would incite you. Vary your study of medicine occasionally by an incursion into the domain of mental philosophy. You will there learn not only the laws of common sense, but numerous others, whose friendly light shall chase the gloom from many an intricate pathway in your future career. The records of medicine abound with examples of men, who, deprived of the advantages of early training in the schools, have, by dint of hard study and shrewd observation, under the auspices of this peculiar genius for medicine, achieved for themselves both fortune and renown.

The famous Dr. Radcliff, of London, was once asked by a young man what book he should read in order to become a physician. "Go," answered the eccentric but gifted Doctor, "go study Don Quixote." It is not related whether the advice was followed or not, but I venture the assertion that the inquirer failed to comprehend the valuable philosophy concealed in the remarkable reply of the great practitioner. Books and learning are proper and essential, but unsupported by medical sense and the inductions of experience, they can bear no valuable fruit.

It was another celebrated physician of London who, being visited one day by a man who naturally enough associated so famous a doctor with a great library, asked him where was his library. The doctor, pointing significantly to a pile of loose bones of the human skeleton lying in a window casing, said, "There, sir, is my library."

A few years ago, in a neighboring state, it was frequently my fortune to meet a poor fellow, whose characteristics as a physician, sad as was his fate, remind me of the principle I am inculcating. He was a plain, unassuming man, and evidently of meagre culture in any early school or home. To a stranger his dress and general appearance indicated anything else than the intelligent physician; and, worse than all, he was a hopeless inebriate. Yet this man, when sober and perfectly at himself, seemed gifted with a sort of inspiration at the bedside of the sick. By a mental process, whose rapidity of analysis and deduction was as remarkable as its sagacity and ability, he arrived at his diagnosis and plan of treatment. The people among whom he practised regarded him as another Ambrose Pare. Poor fellow! like too many others of his profession, he deliberately and apparently bereft of all strength to resist the terrible temptation, sacrificed talents which, properly used, would have raised him to wealth and distinction.

Cultivate with all diligence, therefore, both the spirit and methods of philosophy; not that of Plato and Aristotle, but the philosophy of Bacon, of Harvey and Jenner. Like Socrates, however, in one respect I would have you to be; like him be not ambitious of originating a system. Observe faithfully, accumulate facts, and be sure that they are facts; discover and defend truth, and in due time, without bigotry or bitter wrangling, the true theory of medicine, as a science and an art, will find full expression in the general intelligence and improved methods of the times.

If any one feature more than another marks this era it is a spirit of patient, cautious, rigorous scientific induction. Every assumption, whether truthful or no, is subjected at once to the crucial test. It is an age, too, both formative and transitional; ancient landmarks are fast fading from view, to give place to the novel formations of yesterday. Whole mountain piles of effete knowledge and theory are vanishing away, while new forces, we trust of truth, are shooting up lofty peaks in their stead. No student of medicine can afford to be idle now and hope to rise in his profession. He must needs be a student, a philosopher, or nothing. It is obvious that the mission of this generation of physicians is to accomplish a large part of the colossal work which awaits performance, preparatory to that grand future triumph of medicine to which all the signs of the times are so hopefully pointing.

It has been asserted by a distinguished thinker, himself a physician, that there are more false facts than false opinions in medicine. If this be true, then it is only the conservative influence of that sterling medical sense, which I have described as eminently characteristic of English and American physicians, that can at all account for this remarkable anomaly, and save the profession from the deeper odium of general malpractice. No system can be truly scientific that abounds in false facts. If the facts are false the induction is false, and thus far the whole fabric is false. But all the facts of medicine are not false; yet while so many are, it most resembles the magnificent image of the Babylonian monarch's dream, whose beautiful proportions were composed partly of gold and partly of untempered clay. This is mingled weakness and strength, but development and not demolition is here the law of its renovation. It awaits the touch of the magician's wand, who is to bring order out of confusion, dignity out of weakness, and transmute the entire image into the beauty and strength of homogeneous gold.

The spirit of progress in any department of knowledge is always more or less contagious. Distinguished advancement in one is a sure pledge of a corresponding advancement in all the rest; coming events are casting, not shadows, but light, before them, and it is impossible not to discern in the signs around us that we are standing upon the very eve of glorious issues for the science of medicine. I say eve, but it may be, in the nature of things, an eve of long twilight, of hope deferred; for you must not forget the vast multitude of objects and effects and causes and complications involved in the mighty problem. I only mean to assert that the sun of its triumph will surely rise, and that now at no very distant period.

Each of you, I trust, will go forth to perform an important part in the consummation of this great work. Keep in memory the favorite maxim of Aristotle: "Regard nothing as knowledge that does not explain the causes of things," and you cannot fail to make many a valuable addition to the fund of genuine acquisitions of the profession. Accept nothing on mere authority; submit it to the test of your own experience and legitimate experiment. If at any time you must needs be empirical, only be so with an earnest mental protest.

One of the very first practical lessons which you will be required to observe in your professional life will be to think for yourselves. Indeed, you cannot travel far with your budget of cut-and-dried knowledge of the books before discovering that the curious fact in optics, that no two observers see the same rainbow, is substantially true, as well, for other objects and in other departments of natural science. You will find that neither patients, nor diseases, nor symptoms, not having studied medicine, will always hold and behave themselves just as the books say they ought. They will often take it into their heads to go off at a sad tangent to the old beaten circular path, and the best you can do under such circumstances will be to lighten yourselves at once of the burden and trammels of the books, and go in pursuit disencumbered and trusting alone to your own eyes and spontaneous resources. A man who can neither see nor think for himself will never make a physician; but if to these disqualifications he add a large fund of audacity, he may become a prince of quacks. Such was Joe Smith in religion and Helmbold in medicine.

But if I were asked to define the highest essential attribute of the physician, I would unhesitatingly answer, *heroism*. No man can be a great physician—a Napoleon in his profession—without a large proportion in his mental and physical constitution of the heroic. Self-reliance is the chief element in the character of the hero, and no profession, not even that of the military commander, demands a larger fund of it than that of medicine. The first and last lesson of the physician is self-reliance. Despite learning and books and prestige, when his self-reliance fails him, he is lost. But this is the requirement of only his every-day life; at any moment he may be called into situations where his self-reliance must rise into the moral sublime, the heroic, or he is equally lost. No two illustrious men of the profession possessed more of this valuable attribute than Dupuytren and the late lamented Nelaton, of France.

A short time before his death Nelaton was called to attend the Prince Imperial in a deep-seated abscess of the hip-joint. Self-controlled as Louis Napoleon was constitutionally, the slightest illness of the Prince always unmanned him. Nelaton gave the case a careful examination, and decided, with his usual coolness, upon his treatment. He resolved to lay open the abscess. The Emperor, nervous and excited, objected. "But it must be done," insisted Nelaton; "his life depends upon the operation." "Nelaton," replied the Emperor, "I see no abscess there, and not the least indication that the knife is necessary. You will kill the Prince." "Will your Majesty leave the case with me?" insisted Nelaton. "The Prince will die if he is not relieved of this abscess." Napoleon then reluctantly consented, and, turning round to a window in the apartment, leaned his head upon the sash. Nelaton hastened to use his bistoury, but on the withdrawal of the instrument not a drop of pus appeared, but blood flowed copiously from the wound. The Emperor became furious, and rushing towards the surgeon, exclaimed frantically; "Did I not tell you that no abscess was there, and that you would murder my child?" The situation was now appalling; but Nelaton stood calm and unmoved. He had formed his diagnosis, and knew what he was doing. Quickly eluding the grasp of the Emperor, he in an instant plunged his bistoury a second time into the deep-seated abscess, whereupon, the pus having been reached, escaped, to the relief of the patient, and the complete vindication of the heroism and professional sagacity of the great surgeon.

In conclusion, gentlemen, I would earnestly commend to your study the biography of the illustrious men whose achievements and home-life have adorned the profession of medicine. There is no department of English or American history more brilliant for intellectual activity, or more heroic for self-sacrifice in the cause of truth and humanity, than the lives of our great physicians and surgeons. From Rush and John Hunter catch the inspiration of your philosophy. From Jenner and Harvey learn to be truthful and modest, and imbibe from their achievements that spirit of patient investigation, which only can give you success in your professional life. From Cooper and Syng Physick dare to lift your eyes to the ideal surgeon. Above all, be devout with the gentle Good, and like Jenner despise the meanness of mere self-aggrandizement, and the low ambition of living for popular applause.

And then, when your work is done, when the heat, and toil, and sacrifice are past, after a life consecrated to the good of your fellow-men, having kept the faith, and, it may be, crowned with the blessings of those whom you have lived to benefit, in the evening you will lie calmly down, not to die, but to enter upon the fruition of a glorious rest beyond the river.

